# SPI-1 encoded genes of *Salmonella* Typhimurium influence differential polarization of porcine alveolar macrophages *in vitro*

**DOI:** 10.1186/1746-6148-8-115

**Published:** 2012-07-20

**Authors:** Kamila Kyrova, Hana Stepanova, Ivan Rychlik, Martin Faldyna, Jiri Volf

**Affiliations:** 1 Veterinary Research Institute, Hudcova 70, 621 00, Brno, Czech Republic

## Abstract

**Background:**

Within the last decade, macrophages have been shown to be capable of differentiating toward a classically activated phenotype (M1) with a high antimicrobial potential or an alternatively activated phenotype (M2). Some pathogens are capable of interfering with differentiation in order to down-regulate the anti-microbial activity and enhance their survival in the host.

**Results:**

To test this ability in *Salmonella enterica* serovar Typhimurium, we infected porcine alveolar macrophages with wild-type *Salmonella* Typhimurium and its isogenic mutants devoid of two major pathogenicity islands, SPI-1 and SPI-2. The induction of genes linked with M1 or M2 polarization was determined by quantification of gene expression by RT-qPCR. The ΔSPI-1 mutant induced a high, dose-dependent M1 response but a low M2 response in infected macrophages. On the other hand, wild-type *Salmonella* Typhimurium induced a low M1 response but a high, dose-dependent M2 response in infected macrophages. The response to ΔSPI-2 mutant infection was virtually the same as the wild-type strain.

**Conclusions:**

We therefore propose that *Salmonella* Typhimurium DT104 studied here can polarize macrophages towards the less bactericidal M2 phenotype and that this polarization is dependent on the type III secretion system encoded by SPI-1.

## Background

*Salmonella enterica* serovar Typhimurium (*Salmonella* Typhimurium) is a facultative, intracellular pathogen capable of causing severe gastroenteritis in mammals including humans. Although the virulence of *Salmonella* Typhimurium is a multifactorial phenotype, there are two key virulence determinants specific for *S. enterica*. To successfully interact with the immune system of the host, *S. enterica* is equipped with two type three secretion systems (T3SS-1 and T3SS-2) encoded by *Salmonella* pathogenicity island 1 (SPI-1) and *Salmonella* pathogenicity island 2 (SPI-2), respectively. These secretion systems evolved to deliver *S*. *enterica* proteins directly into the cytoplasm of the host cells. T3SS-1 translocates *S*. *enterica* proteins across the cytoplasmic membrane and promotes *S*. *enterica* invasion into non-phagocytic cells [1,2]. T3SS-2 translocates *Salmonella* proteins into the cytoplasm of the host cell across the phagosome membrane and is essential for *Salmonella* survival and replication inside professional phagocytic cells [3].

Macrophages represent the key cells of host defense against *Salmonella* as well as other infections. Within the last decade, macrophages have been shown to have a plastic phenotype, which is dependent on microenvironmental stimuli. Based on gene expression, the macrophages can be divided into two major subtypes; pro- and anti-inflammatory. The pro-inflammatory macrophages (also called M1 polarized or classically activated) comprise of inflammatory cells developed under stimulation by LPS and IFNγ. Such macrophages exhibit high phagocytic and antimicrobial activity and high expression of pro-inflammatory cytokines (e.g. TNFα, IL-8, IL-12, IL-23, IL-1) [4]. In contrast, the alternatively activated macrophages (also called M2 polarized) represent a phenotypically less homogenous group with a low phagocytic and bacterial killing ability, low expression of pro-inflammatory cytokines and high expression of non-phagocytic receptors. M2 polarized macrophages play an important role in the reparative phase of inflammation but they are also found in connection with parasitic and chronic bacterial infections. Several gene expression studies have shown that macrophages commonly follow M1 polarization in response to infection with a broad spectrum of Gram-negative and Gram-positive bacteria [5,6]. However, some bacterial pathogens are able to manipulate macrophage gene expression and induce the M2 program in order to escape the hostile environment present in M1 polarized macrophages [6-8].

In our previous study, we showed that the absence of the SPI-1 T3SS system led to a significantly higher pro-inflammatory response of porcine alveolar macrophages (PAM) not only at mRNA but also at protein level [9]. This indicated that SPI-1 encoded T3SS might be involved in the suppression of M1 macrophage polarization. However, we did not address to what extent the number of intracellular bacteria influences such signaling and we also did not determine the expression of genes characteristic of M2 macrophage polarization. In the present study, we therefore focused on the expression profile of the anti-inflammatory response genes in macrophages in response to *Salmonella* Typhimurium infection and the relationship of macrophage expression to the number of intracellular bacteria.

## Methods

### Bacterial strains and growth conditions

*Salmonella* Typhimurium 16E5 belongs to phage-type DT104 and its isogenic ΔSPI-1 and ΔSPI-2 mutants were used in the study. *Salmonella* Typhimurium ΔSPI-1 and ΔSPI-2 mutants were constructed using the one-step lambda red recombination of PCR products [10] removing the whole pathogenicity island 1 or 2. The deletion of SPI-1 comprises removing 36 genes from avrA up to invH, all of which are known or predicted to be related to T3SS-1. The deletion of SPI-2 comprises removing 44 genes including orf48, orf32, orf245, orf408, ttrACB and the whole T3SS-2 up to the ssaU gene [11]. Before macrophage infection, overnight cultures were diluted 500 × in LB broth and incubated for 6 h at 37 °C to reach late logarithmic stage culture with maximally expressed SPI-1 genes [12]. Just before infection the bacteria were washed and re-suspended in sterile PBS to OD = 0.3. Heat inactivated bacteria of wild-type and ΔSPI-1 mutant were prepared by heating the culture to 65 °C for 30 min.

### Isolation and cultivation of PAMs

PAMs were obtained from the lungs of clinically healthy pigs immediately after slaughter by sterile bronchoalveolar lavage as described previously [9]. Isolated PAMs (approx. 2 × 10^5^ of PAMs per well of 24-well microplate) were allowed to attach for 2 h in DMEM (Gibco, USA) supplemented with antibiotics (penicillin 100 U/ml; streptomycin 100 μg/ml, and gentamicin 4 μg/ml). Two hours later, porcine serum (Invitrogen, USA) was added to a final concentration of 10%. After 16-hours of cultivation, just prior to the infection with *Salmonella*, the non-adherent cells were washed away and the medium was replaced with DMEM supplemented with 10% porcine serum and free of any antibiotics. The porcine serum was inactivated (56 °C, 30 min) and was free of anti-*Salmonella* antibodies (tested by Salmotype Pig Screen ELISA, Labor Diagnostik, Germany).

Macrophage cell death was determined by release of LDH using a commercial kit (Promega, CytoTox 96 Non-Radioactive Cytotoxicity Assay) according to the instructions of the manufacturer.

### Experimental infection

To study the effect of PAM infection with *Salmonella* Typhimurium, multiplicity of infection (MOI) 10 was used. However, when the effect of the number of internalized bacteria was analyzed, MOI 2.5 and MOI 40 were used as well*.* One hour after the infection, 100 μg/ml of gentamicin was added to kill any extracellular *Salmonella* Typhimurium. After another hour, the medium with 100 μg/ml of gentamicin was replaced with a fresh one containing a bacteriostatic concentration of gentamicin (15 μg/ml) to prevent extracellular *Salmonella* Typhimurium replication. *Salmonella*-PAM interaction was terminated 24 hours after the infection when the lysates of the cells in RLT buffer (RNeasy kit, Qiagen) were harvested. The numbers of intracellular *Salmonella* in PAMs 4 hours post infection were determined after lysis with 1% Triton X-100 for 20 min. The suspensions were then serially diluted and plated on LB agar. Heat killed bacteria of wild-type and ΔSPI-1 strains were used as controls at MOI 10 and 40 and LPS at a concentration of 1 μg/ml. Negative controls included an assay performed with PAMs without any contact with *Salmonella* or LPS.

### Cytokine gene expression determined by RT PCR

To verify the consistency of results with the previous study [9], three pro-inflammatory cytokines (TNFα, IL-8 and IL-1β) were measured. To test NFκB involvement in this response, NFκBIα was measured as a part of NFκB direct regulation because this gene is under tight transcriptional control of NFκB [13]. M2 polarization genes were selected based on previous reports. These included IL-4R [14], mannose receptor and CD163 [15-17]. Transferrine receptor 1 (TfR1) [15], tissue inhibitor of metalloproteinases 1 (TIMP-1) [18], tissue metalloproteinases MMP9 and MMP12 [19] were the other genes that were chosen based on their high expression in murine or human M2 polarized macrophages. Bruun et al. [19] also report an increase in expression of arginase-1 in M2 macrophages. Finally, IL-10 was measured as the anti-inflammatory cytokine. Hypoxanthine phosphoribosyl transferase I (HPRT) mRNA was chosen as the most stable house-keeping reference gene based on Genorm analysis [20]. The other tested genes which showed less stabile transcription were: beta-2-microglobulin, glyceraldehyde-3phosphate dehydrogenase; histone H3; hydroxymethyl-bilane synthase; succinate dehydrogenase complex, subunit A; TATA box binding protein and phospholipase A2.

Total RNA was purified using the RNeasy kit (Qiagen, Germany) according to the manufacturer’s instructions. The quantity and quality of RNA was checked spectrophotometrically and by agarose gel electrophoresis. Purified RNA was reverse transcribed with M-MLV reverse transcriptase (200 U, Invitrogen) and oligo-dT primers, and the cDNA was used either immediately or was stored at – 20 °C until use. Primers IL-1β, TNFα and HPRT were adopted from [21] and IL-8 primers were designed according to [22]. The remaining primers were designed using Primer3 software [23] and all the primers are listed in Table 1. RT-PCR was performed with QuantiTect SYBR Green PCR Kit (Qiagen) using the LightCycler 480 (Roche). Expression levels of target genes were determined as follows. The threshold cycle values (C_t_) of gene of interest were first normalized to the C_t_ value of HPRT reference mRNA (ΔC_t_) and the normalized mRNA levels were calculated as 2^(−ΔC^_t_^)^.

**Table 1:** List of primers used for RT PCR quantification of gene expression

	**Sequence 5'-3'**	**Reference**
CD163-For	CTTGGGGCAGCGTTGGCAGGAATAG	this study
CD163-Rev	ATGCAGGGCTGATGTCCCCTCTGTC	this study
HPRT-For	GAGCTACTGTAATGACCAGTCAACG	[21]
HPRT-Rev	CCAGTGTCAATTATATCTTCAACAATCAA	[21]
IL10-For	TGAAGAGTGCCTTTAGCAAGCTC	this study
IL10-Rev	CTCATCTTCATCGTCATGTAGGC	this study
IL1β-For	GGGACTTGAAGAGAGAAGTGG	[9]
IL1β-Rev	CTTTCCCTTGATCCCTAAGGT	[9]
IL4R-For	TTCAACACTGAAAACCACACCAC	this study
IL4R-Rev	GTGTCCACAATGACAATGCTCTC	this study
IL8-For	TTCTGCAGCTCTCTGTGAGGC	[22]
IL8-Rev	GGTGGAAAGGTGTGGAATGC	[22]
MannR-For	GACCAAAAATTGTTGATGCTGA	this study
MannR-Rev	GCACCCGTTAGAATCAGGAG	this study
MMP12-For	AGAGGAGGCACATCATGGAC	this study
MMP12-Rev	CTTCTGGTGACACGATGGAA	this study
MMP9-For	CCTTGAACACACACGACATCTTC	this study
MMP9-Rev	CCACATAGTCCACCTGATTCACC	this study
NFκBIα-For	ACGAGCAGATGGTGAAGGAG	this study
NFκBIα-Rev	TCATGGATGATGGCCAAGT	this study
TFRC-For	AATTCAGAGTTGACATAAGGGAGATG	this study
TFRC-Rev	AGACGTAGCACGGAAGAAGTCTC	this study
TIMP1-For	CAGGAGTTTCTCATAGCTGGACAAC	this study
TIMP1-Rev	GTTCCAGGGAGCCACAAAACT	this study
TNFβ-For	CCCCCAGAAGGAAGAGTTTC	[21]
TNFβ-Rev	CGGGCTTATCTGAGGTTTGA	[9]
Arginase1-For	CCAGTCCATGGAGGTCTGTC	this study
Arginase1-Rev	GTGTCTTCCCCAGAGATGGA	this study

### Statistical analysis

Data are presented as the mean and standard deviation of results measured from macrophages originating from 8 to 16 pigs. However, as the experiments were performed with independent batches of PAMs originating from outbred pigs, we commonly observed high and low responders. To normalize the data among different macrophage batches, the transcription rates of individual genes were therefore divided by the arithmetical mean of each individual experiment consisting of macrophages infected with wild type *Salmonella*, SPI1 mutant each at MOI = 10, LPS and non-stimulated cells. The normal distribution of data was tested and subsequently, one-way ANOVA followed by Tukey’s post-hoc test (Prism, Graph Pad Software, La Jolla, USA) were used to compare the differences in transcriptional levels. Significant differences were defined as those with *p* < 0.05.

## Results

### Invasion and cytotoxicity

**Figure 1:**
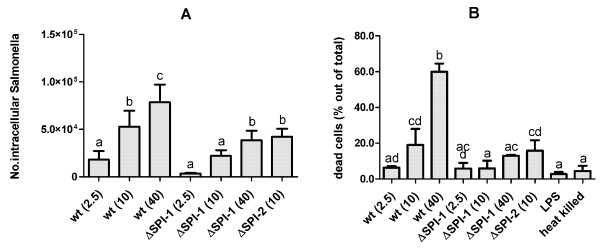
**Invasion and cytotoxicity of*Salmonella*Typhimurium for porcine alveolar macrophages.** Panel **A**, invasion of the wild-type *Salmonella* Typhimurium (at specified MOI) and isogenic ΔSPI-1 and ΔSPI-2 mutants into porcine alveolar macrophages 4 h post-infection. Panel **B**, cytotoxicity of *Salmonella* Typhimurium and its isogenic mutants for porcine alveolar macrophages 24 h post-infection. The data are presented as mean and SD of percentage of LDH released following particular stimulation relative to LDH released from PAMs lysed by freeze-thaw disruption (100%). Significantly different groups (*P* < 0.05) are marked by different letters (“a” differs from “bc”, but not “ab”, etc.). Data are presented as mean and SD.

To investigate how the presence of active SPI-1 and SPI-2 T3SS affects the early gene expression of porcine macrophages *in vitro*, PAMs were infected with wild-type *Salmonella* Typhimurium and the isogenic ΔSPI-1 or ΔSPI-2 mutants. The number of intracellular bacteria was dependent on the presence of SPI-1 T3SS as the counts of the ΔSPI-1 mutant were 2.7 fold lower than the counts of wild-type strain. Counts of the intracellular ΔSPI-2 mutant did not statistically differ from wild-type *Salmonella* Typhimurium. When different MOI were used, the numbers of intracellular bacteria increased with increasing MOI although intracellular counts of the ΔSPI-1 mutant were always lower than the counts of wild-type *Salmonell*a Typhimurium (Figure 1A).

The level of cytotoxicity of bacteria 24 hours post infection was measured by the activity of LDH released from the dead cells into the culture media. Macrophage cytotoxicity of the wild-type *Salmonella* Typhimurium did not differ from that of the ΔSPI-2 mutant but both these strains were more cytotoxic when compared to the macrophages infected with the ΔSPI-1 mutant. In addition, the cytotoxicity of wild-type *Salmonella* Typhimurium increased with increasing MOI while the ΔSPI-1 strain did not (Figure 1B).

### M1-related signaling at MOI 10

Similar transcriptional patterns were observed for IL-1β, IL-8, TNFα and NFκBIα, i.e. the PAMs responded by a higher expression when infected with the ΔSPI-1 mutant than with the wild-type strain or the ΔSPI-2 mutant. All these differences disappeared when the PAMs were stimulated with heat killed bacteria of both wild-type *Salmonella* Typhimurium or the ΔSPI-1 mutant.

**Figure 2:**
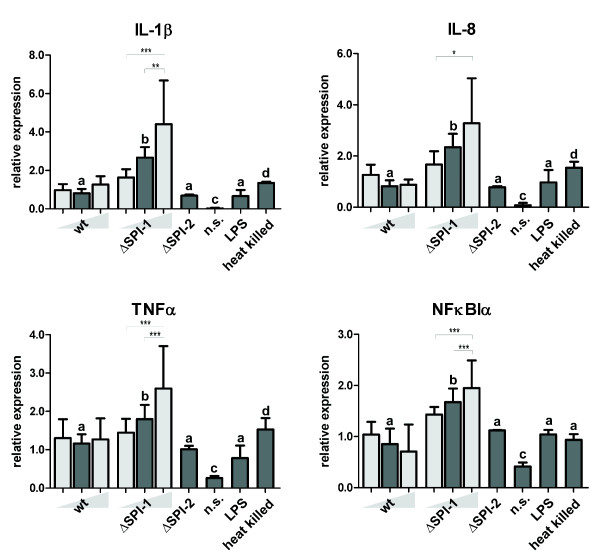
**M1-related gene expressions.** Dark grey columns represent M1-related gene expression in PAMs 24 hours post infection with the wild-type *Salmonella* Typhimurium, ΔSPI-1 and ΔSPI-2 mutants using MOI = 10. Stimulation with LPS from *Salmonella* Typhimurium (1 μg/ml), heat killed *Salmonella* Typhimurium and non-stimulated cells (n.s.) were used as controls. Significantly different groups (*P* < 0.05) are marked by different letters (“a” differs from “bc”, but not “ab”, etc.). Light gray columns represent data for different MOI (=2.5 and 40) of wt strain and ΔSPI-1 mutant. The statistical significances are marked only among the groups infected with different MOIs of the same strain. The significance levels are as follows 0.05 (*), 0.01 (**), and 0.001 (***). Data are presented as mean and SD.

Except for NFκBIα, heat-killed bacteria stimulated a higher response in PAMs than those induced by wild-type *Salmonella* Typhimurium and the ΔSPI-2 mutant but lower than those induced by the ΔSPI-1 mutant. Purified LPS stimulated pro-inflammatory signaling of PAMs to the same extent as the wild-type strain or the ΔSPI-2 mutant (Figure 2).

### M1-related signaling at variable MOI

Since the behavior of the wild-type strain and the ΔSPI-2 mutant did not differ, the dose dependent experiments were performed only with the wild-type *Salmonella* Typhimurium and the ΔSPI-1 mutant. The transcription of M1-related genes did not correspond to a lower or higher MOI of wild-type *Salmonella* Typhimurium as no significant differences were observed in signaling of PAMs infected at MOI 2.5, 10 or 40 (Figure 2) despite the fact that the numbers of intracellular *Salmonella* correspondingly increased. On the other hand, the transcription of the same genes showed a clear dose-dependent profile in PAMs infected with the ΔSPI-1 mutant. Stimulation of PAMs with heat killed wild-type *Salmonella* Typhimurium and the ΔSPI-1 mutant at MOI 40 did not result in the induction of any M1 related genes when compared with experiment with MOI 10 (data not shown).

### M2-related signaling at MOI 10

**Figure 3:**
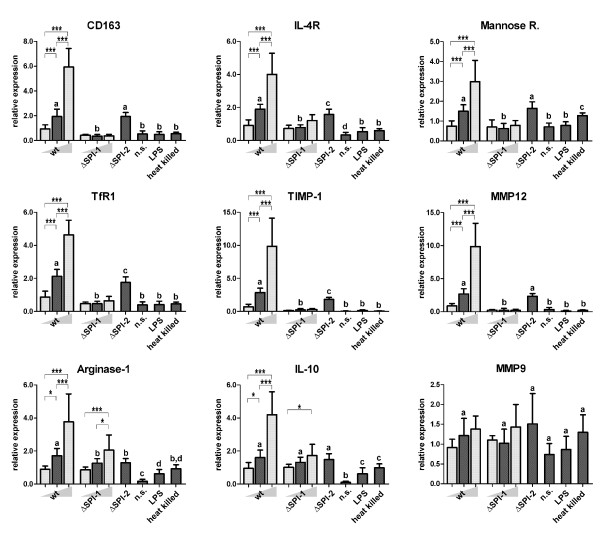
**M2-related gene expressions.** Dark grey columns represent M2-related gene expression in PAMs 24 hours post infection with the wild-type *Salmonella* Typhimurium, **Δ**SPI-1 and **Δ**SPI-2 mutants using MOI = 10. Stimulation with LPS from *Salmonella* Typhimurium (1 μg/ml), heat killed *Salmonella* Typhimurium and non-stimulated cells (n.s.) were used as controls**.** Significantly different groups (*P* < 0.05) are marked by different letters (“a” differs from “bc”, but not “ab”, etc.). Light gray columns represent data for different MOI (=2.5 and 40) of wt strain and **Δ**SPI-1 mutant. The statistical significances are marked only among the groups infected with different MOIs of the same strain. The significance levels are as follows 0.05 (*), 0.01 (**), and 0.001 (***). Data are presented as mean and SD.

Except for three genes, most of the genes showed a similar transcriptional pattern. These 3 exceptions included MMP9 which was not induced by any stimulation, IL-10 which transcription did not differ following infection with all three strains used, and arginase-1 which was slightly higher induced by the wild-type than the ΔSPI-1 or ΔSPI-2 mutants. The genes with the characteristic pattern for M2-related response comprised IL-4R, mannose receptor, TfR1, CD163, MMP12 and TIMP1 and in all of them, significantly lower expression was observed after PAM infection with the ΔSPI-1 mutant when compared to the expression in PAMs infected with wild-type Salmonella Typhimurium or the ΔSPI-2 mutant (Figure 3).

As in the case of M1-related signaling, the differences between the wild-type strain and the ΔSPI-2 mutant were mostly insignificant with only a few exceptions (IL-4R, TfR1, TIMP1 and arginase-1). However, even in such cases, the differences were minor when compared to those induced by the ΔSPI-1 mutant. Unlike the pro-inflammatory genes, the M2-related genes were not induced in PAMs exposed to heat killed bacteria of wild-type *Salmonella* Typhimurium and ΔSPI-1 or LPS.

### M2-related signaling at variable MOI

The PAM anti-inflammatory response to infection with the wild-type *Salmonella* Typhimurium and the ΔSPI-1 mutant was the opposite of that observed for the pro-inflammatory genes. The transcription of anti-inflammatory genes exhibited a clear dose-dependent profile after infection with the wild-type strain (Figure 3). On the other hand, the increase in MOI of the ΔSPI-1 mutant did not induce M2-related genes expression as no significant differences were observed in signaling of the PAMs infected at MOI 2.5, 10 or 40.

## Discussion

In this study, we have shown that the presence of intact SPI-1 genes enables *Salmonella* polarization of PAMs towards the less bactericidal M2-related response. This conclusion is supported by the fact that the M2 signaling exhibited a dose-dependent response after infection with the wild-type *Salmonella* Typhimurium whilst M1 signaling was dose independent. In fact, the M1 signaling after infection with the wild-type *Salmonella* Typhimurium appeared to be a mere response to LPS since the LPS response, response to heat killed *Salmonella* Typhimurium and responses to the wild-type strain at different MOI were all similar (Figure 2). On the other hand, when SPI-1, but not SPI-2, genes were removed from the *Salmonella* Typhimurium chromosome, such a mutant was unable to modify macrophage polarization towards the M2. Indeed, infection of PAMs with the ΔSPI-1 mutant resulted in high, MOI dependent, pro-inflammatory signaling. Simultaneously, the SPI-1 mutant was essentially unable to induce the expression of M2 related genes, regardless of the MOI used.

Except for MMP9, for which transcription did not react to any kind of stimulation, the transcriptional profiles of the M2 related genes are clustered into two groups. The first group included IL-10 and arginase-1. These two genes were induced by both strains in a dose-dependent manner regardless of the presence of SPI-1. This can be explained by the fact that, although these genes are related to M2 [7], IL-10 can be activated by the NF-κB signaling pathway in the late phases of infection to quell the immune response [24] and arginase-1 is induced following toll like receptor stimulation [25]. These two genes therefore partially share signaling pathways with the pro-inflammatory genes. The remaining group of mannose receptor, CD163, TfR1, TIMP-1, IL-4R and MMP12 exhibited a highly similar transcriptional pattern. As this pattern was exactly the opposite of that seen for M1 related genes, we propose these genes belong among the M2 polarization markers in PAMs.

The suppression of pro-inflammatory signaling is macrophage specific. The pro-inflammatory signaling in other cells is dependent on *Salmonella* invasion, i.e. it is dependent on intact SPI1 [21]. Interestingly, the alternative function of SPI1 genes in relation to differential cytokine signaling may have a clear evolutionary advantage. It has been shown recently that the inflammation induced by *Salmonella* invading tissue results in host responses which either limit the growth of bacteria different from *Salmonella*[26] or result in oxidation of intestinal metabolites, which can be used by *Salmonella* for efficient proliferation at the mucosal surfaces [27]. The invading *Salmonella* cells thus provide the noninvasive part of the *Salmonella* population a growth advantage in the gut mucus over the rest of the mucosal microbiota. However, once *Salmonella* crosses the epithelium and comes in contact with macrophages, bactericidal M1 polarization would result in *Salmonella* eradication and a decrease of pro-inflammatory signaling by the epithelial cells. In addition, M2 polarized macrophages may act against the signaling of epithelial cells and keep an appropriate balance between the epithelial cells and macrophages, which may result in the optimal production of *Salmonella* required metabolites and limited damage to the host. Interestingly, the ability of *Salmonella* to polarize the macrophage response towards the M2 might be further potentiated in host adapted serovars. In such cases, this polarization may enable *Salmonella* to escape macrophage killing and, instead, these serovars can be distributed inside the macrophage across the host’s body without being effectively recognized and inactivated.

## Conclusions

All of the presented results support the hypothesis that *Salmonella* Typhimurium DT104, similar to other bacterial pathogens [6-8], can modify macrophage polarization from the pro-inflammatory and bactericidal M1 rather towards a non-inflammatory M2 and that this polarization is dependent on an intact SPI-1.

## Competing interests

The authors declare that they have no competing interests.

## Authors’ contributions

KK contributed to the data collection and analysis, laboratory work, drafting and writing of the manuscript. HS contributed to the design of the study, laboratory work, and drafting of the manuscript. IR and MF contributed to the conception, the acquisition of funds, supervision and drafting of the manuscript. JV contributed to the conception, design, data analysis, laboratory work, drafting and writing of the manuscript. All authors have read and approved the final manuscript.
